# Osteosarcoma: current insights and advances

**DOI:** 10.37349/etat.2025.1002324

**Published:** 2025-06-15

**Authors:** Guraustin S. Brar, Aidan A. Schmidt, Logan R. Willams, Mark R. Wakefield, Yujiang Fang

**Affiliations:** IRCCS Istituto Romagnolo per lo Studio dei Tumori (IRST) “Dino Amadori”, Italy; ^1^Department of Microbiology, Immunology & Pathology, Des Moines University, West Des Moines, IA 50266, USA; ^2^Department of Surgery, University of Missouri School of Medicine, Columbia, MO 65212, USA; ^3^Ellis Fischel Cancer Center, University of Missouri School of Medicine, Columbia, MO 65212, UAS

**Keywords:** Osteosarcoma, gene therapy, immunotherapy, radiation, tyrosine kinase inhibitors

## Abstract

Osteosarcoma is the most prevalent primary malignant bone tumor affecting adolescents and young adults. Despite advancements in cancer therapies, its prognosis remains poor due to its aggressive nature and early propensity for metastasis—often present at the time of diagnosis. The etiology of osteosarcoma is multifactorial, involving genetic predispositions, environmental exposures, and familial syndromes. While treatment strategies are largely dictated by tumor stage, neoadjuvant chemotherapy followed by surgical resection remains the cornerstone of management. This review provides a comprehensive overview of osteosarcoma, including its historical context, subclassifications, clinical presentation, diagnostic approaches, and evolving treatment modalities. Recent therapeutic innovations—such as gene therapy, immunotherapy, radiation advances, and tyrosine kinase inhibitors—are discussed in detail, highlighting their mechanisms and clinical potential. By synthesizing current literature and identifying ongoing challenges, this review aims to inform clinicians and researchers of recent progress while highlighting critical gaps to guide future research and improve patient outcomes in osteosarcoma care.

## Introduction

Osteosarcoma (OS), first described by Alex Bowyer in 1805, is a proportionately rare form of cancer, making up around 1% of total cancer cases worldwide, and is commonly seen in adolescents and young adults, with an incidence of 3.4 per 1 million [1]. OS is a highly aggressive bone malignancy that can arise in any skeletal region. However, it predominantly manifests in the long bones, with a tendency to affect areas directly surrounding the knee joint [2]. Histologically, OS originates from mutated mesenchymal cells, which are undifferentiated multipotent cells capable of differentiating into a wide array of cell types. Through the transformation process, these mesenchymal cells encounter replication errors and form into malignant osteoblasts, which classifies them under the category of OS [3]. These mutant osteoblasts exhibit uncontrolled production of osteoid and aggressive proliferation within the affected bone. The proliferation rate of mutated osteoblasts is often elevated to levels 10 to 20 times greater than physiological baseline [4]. Such rapid growth leads to the accumulation of osteoblastic mass and subsequent tumor formation due to a high frequency of errors during bone formation [5]. In addition to cancerous formation at the site of the affected bone, OS also poses a significant risk of metastasis. When OS metastasizes, it most commonly spreads to the lungs, although infiltration into the lymphatic system has been noted in some patients [6]. The metastatic process involves the infectious OS cells from the primary tumor site to be transported to another location in the body to establish a secondary tumor site. The primary route of OS spread is through direct access to the bloodstream or lymphatic system [7]. Such metastatic spread significantly complicates patient treatment and remains a major determinant of overall patient outcomes. The overall survival rate for OS is highly dependent on metastasis status and the disease subclass [8].

## Subclasses

OS has the ability to infiltrate various skeletal regions, which allows for it to be further classified based on specific histological and microbiological anomalies. This classification enables a more precise understanding of the cancer’s behavior and disease progression. The two primary subclasses of OS are central and surface, both showing characteristic features in origin, growth, and progression [9].

### Central (intramedullary) OS

Central, or intramedullary OS, represents the most prevalent form of OS, predominantly arising within the bone marrow. It is often localized at the metaphyseal growth plates, which are located on the distal ends of long bones, such as the arms and legs [10]. From a microbiological perspective, central OS arises from aberrations in osteoblasts, the specialized cells that play a critical role in regulating bone growth and development. Such abnormalities lead to excessive bone matrix production from within the bone, disrupting normal development and contributing to the onset and progression of the disease state [11]. Mutations in TP53, a common tumor suppressor that integrates various stress signals and initiates DNA repair, are also frequently seen in patients with central OS. Moreover, mutations in *RB1* and *CDK4* can both lead to dysregulation of cellular division. *RB1* mutations disrupt cell cycle checkpoints, and *CDK4* mutations result in unchecked G1–S phase transition, both contributing to oncogenesis. However, such mutations are less prevalent than TP53 mutations [12].

### Surface (peripheral) OS

Surface OS, also known as peripheral OS, is the second most common type of OS. Unlike central OS, surface OS typically manifests on the outer surface of bones, appearing as an ossified mass. It is often found on the lower shaft of the femur and is generally considered less aggressive than other forms of the disease [13]. Parosteal OS is the most common subtype of surface OS. It originates from the outer layer of the periosteum, the outermost connective tissue layer of the bone shaft. Due to its superficial location and slow rate of progression, parosteal OS is associated with better patient outcomes compared to other subtypes [14]. The second subtype is periosteal OS, which arises from the inner layer of the periosteum. This variant tends to have a more aggressive clinical course and is associated with a poorer prognosis compared to parosteal OS [15]. The final subtype, high-grade surface OS, is the most aggressive form and carries the highest risk of metastasis. It originates on the periosteum but can quickly invade the underlying bone and adjacent soft tissues at a rapid rate [16].

## Diagnostics

The diagnosis of OS requires a multidisciplinary approach, integrating clinical evaluation, imaging, and laboratory testing. Patients often present with persistent bone pain that worsens at night, along with swelling or a palpable mass [17]. If the tumor is located near a joint, restricted mobility may be observed. X-rays typically reveal a “sunburst pattern”, indicative of aggressive bone lesions [18]. MRI is used to assess local invasion, while CT and bone scans help evaluate metastatic spread. Elevated alkaline phosphatase levels can support the diagnosis by indicating increased bone turnover [19]. A definitive diagnosis is made through biopsy and histopathological analysis. Staging is then determined based on tumor size, metastatic involvement, and histological grade to guide treatment planning.

## Treatment history

Since Alex Bowyer’s discovery of OS in 1805, treatment methods have undergone significant advancements over the past two centuries. Historically, until the mid-19th and early 20th centuries, amputation of the affected limb was the standard treatment. However, due to limited medical technology and poor sanitation, patients who underwent amputation faced a high risk of sepsis and other surgical site infections [20]. As surgical techniques improved in the early 20th century, postoperative infections decreased; however, mortality rates remained high due to the continued risk of metastasis. In the early to mid-20th century, radiotherapy gained popularity, although it initially showed limited effectiveness for OS due to difficulties in accurately targeting cancerous cells [21]. By the 1970s, the gold standard of care had become multifaceted. The introduction of multi-drug chemotherapy significantly enhanced tumor reduction and quickly became the most widely adopted treatment strategy [22]. Chemotherapy allowed for sufficient tumor shrinkage, enabling the development of “limb-sparing surgery”, which soon became a common alternative to full amputation [23]. This approach allowed many patients to retain their limbs, with only minimal removal of bone tissue. In the 21st century, the most common treatment protocol involves neoadjuvant therapy followed by surgical excision of the tumor [24]. Common therapeutic agents include cisplatin, doxorubicin, and methotrexate, all of which target key molecular pathways critical for tumor cell survival and proliferation [25].

## New treatments

### Gene therapy

With ongoing advancements in medical science, emerging therapies for OS demonstrate significant potential. At the forefront of these novel treatment modalities is gene therapy, which works by targeting specific genetic and molecular factors that drive cancer growth and metastasis [26]. Common targets include tumor suppressor genes, pathways involved in drug sensitization, and gene-editing systems such as CRISPR/Cas9, which facilitate the repair of damaged DNA segments.

Gene therapy directed at tumor suppressor genes enables the introduction of functional gene copies into the host genome, helping to restore normal cell cycle regulation and physiological apoptosis [27]. One commonly altered tumor suppressor gene in OS is TP53, whose mutation disrupts the G1 cell cycle checkpoint. This not only promotes tumor growth but also increases the tumor’s reliance on the G2 checkpoint to maintain DNA integrity [28].

Gene therapies aimed at modifying drug sensitization pathways are also showing promise, as many OS cells acquire mutations or express proteins that enhance drug resistance. A major contributor to this resistance is the exosome—small extracellular vesicles normally involved in cell-to-cell communication and environmental modulation. In cancer cells, exosomes can excrete chemotherapeutic agents like doxorubicin, transfer or upregulate drug efflux pumps such as P-glycoprotein (P-gp), and carry multidrug-resistant mRNA from resistant to sensitive cells, facilitating the spread of resistance [29]. Interestingly, the same mechanisms that make exosomes effective vehicles for resistance also make them promising targets for gene therapy. Exosomes possess favorable characteristics such as small size, good solubility, low toxicity, and a long half-life [30]. Programmed exosomes have been explored as delivery systems for miRNA, with studies showing they are often more selective and less cytotoxic than current methods—possibly due to interactions between exosome membrane proteins and tumor cell membranes [29]. A 2022 study demonstrated that exosomes loaded with miR-665 were able to inhibit OS progression both in vivo and in vitro, while maintaining good safety and efficiency profiles [31].

Despite their advantages, exosomes face limitations—primarily the difficulty of isolating them in sufficient quantity and purity, and the lack of data identifying which specific exosomes are most effective across cancer types [29]. Moreover, it remains unclear whether other nanoparticle delivery systems, such as hyper-cell-permeable micelles, may ultimately prove more effective. While exosomes share many beneficial traits, they lack the specificity of lab-engineered nanoparticles [32].

**Figure 1:**
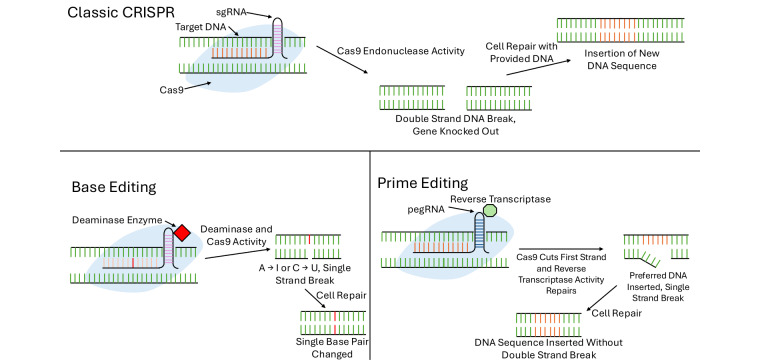
A schematic representation of the CRISPR/Cas9, base editing, and prime editing mechanisms, which compares the differences in components, progress, and outcomes for the three techniques [32, 34]

Gene therapy also enables researchers to leverage CRISPR/Cas9 technology for precise genome editing and the identification of drug resistance genes. This allows for targeted modification of genes contributing to OS development and drug resistance, potentially improving treatment outcomes [33]. CRISPR combats cancer in various ways, with the classic method involving a single-guide RNA (sgRNA) designed to bind a specific DNA sequence, as shown in Figure 1. This RNA guides the Cas9 protein to the target site, where it cuts both DNA strands. The cell then initiates repair mechanisms. CRISPR can be used to inactivate oncogenes, disrupt immune checkpoint genes, or directly induce double-stranded breaks in cancer cell DNA [34]. A 2023 study showed that CRISPR targeting of PLK1—a master regulator of the G2/M checkpoint—successfully knocked out the gene and inhibited OS cell proliferation both in vitro and in vivo [35].

More advanced gene-editing strategies include base editing and prime editing. Base editing enables the conversion of one nucleotide base to another using a Cas9-deaminase fusion, which can convert cytosine to uracil or adenosine to inosine. This process induces repair using the edited strand as a template. While base editing introduces fewer errors, such as translocations or rearrangements, and is ideal for single-nucleotide modifications, it cannot insert DNA fragments or perform gene knockouts, limiting its scope [32].

Prime editing addresses these limitations. It utilizes a prime editing guide RNA (pegRNA), a reverse transcriptase, a Cas9 fusion protein, and a nicking guide RNA to insert indels or point mutations. After binding to the target site and nicking one DNA strand, the reverse transcriptase synthesizes the new sequence, which is copied over once the complementary strand is nicked. This technique offers greater versatility than base editing and higher fidelity than traditional CRISPR, as it avoids double-strand breaks [32]. Although designing pegRNAs remains a challenge, prime editing has already shown success across multiple model organisms and holds promise for personalized medicine.

In addition to direct editing, CRISPR screening is used to identify phenotypes and genes associated with drug resistance. This involves knocking out specific genes and observing cell survival after drug exposure. Surviving cells typically carry resistance-related mutations, which can then be validated across cell lines to determine whether the resistance is drug-specific or generalizable [31]. While gene therapy holds immense promise, further clinical trials are needed to assess long-term safety, delivery efficiency, and therapeutic efficacy before it can receive FDA approval.

### Immunotherapy

Immunotherapy has shown promise in the treatment of OS, particularly in advanced or treatment-resistant cases where conventional therapies often fall short. OS is known for its ability to evade the host immune system, largely through the expression of immune checkpoint proteins such as PD-L1, which suppress immune activity and enable cancer cells to proliferate unchecked [36]. In gastric cancer cells, PD-L1 has been shown to play a significant role in modulating immunosuppression and tumor invasion through stromal cell remodeling, cell signaling, secretion of soluble factors, and immune cell differentiation. It may play a similar role in OS as well [37]. Immunotherapy seeks to counteract this immune evasion by leveraging the host’s immune system to recognize and destroy tumor cells, with a particular focus on reactivating T cells [38]. Agents such as pembrolizumab and nivolumab, which target immune checkpoints like PD-L1 and CTLA-4, are designed to disrupt immunosuppressive signaling cascades, thereby reactivating T cells to mount an anti-tumor response [39]. Nivolumab and pembrolizumab have both been approved as second line treatments for gastric cancer, although more research needs to be done for them to be used for OS, and they were found to be more effective when used in conjunction with other drugs rather than as monotherapy. In gastric cancer, nivolumab was found to have a median overall survival of 5.3 months with placebo at 4.1, while a different study found pembrolizumab to have a median overall survival of 5.6 months [37].

Another promising target for T cell reactivation is TIM-3, a protein expressed on the surface of various immune cells. In the OS tumor microenvironment (TME), TIM-3 binds with Gal-9 to induce apoptosis of CD4^+^ and CD8^+^ T cells. Blocking TIM-3 has been shown to increase both the number and activity of tumor-infiltrating CD8^+^ T cells in OS models [39]. A 2021 phase I/II trial evaluated sabatolimab, an anti-TIM-3 antibody, alone and in combination with spartalizumab, an anti-PD-1 drug, in patients with advanced solid tumors. While sabatolimab alone showed limited efficacy, the combination therapy demonstrated promising activity with minimal adverse effects [40]. Although further research is needed, TIM-3 represents a promising target for future combination therapies.

Chimeric antigen receptor (CAR) T therapy has demonstrated success in hematological malignancies but has shown limited efficacy in solid tumors due to barriers within the TME, such as dense stroma, hypoxic conditions, and immunosuppressive cell populations. CAR T cell therapy involves collecting T cells from the patient or a compatible donor and genetically modifying them to target specific tumor-associated antigens. The basic CAR structure includes an antigen-binding domain, hinge, transmembrane domain, and intracellular signaling domain. Advancements have included the addition of co-stimulatory domains for improved activation, inducible transgenes for safety, and specialized receptors for precise targeting and signaling [41, 42].

Despite these innovations, CAR T cell monotherapy remains largely ineffective in solid tumors like OS. A 2024 phase I trial investigated a GD2-targeted CAR T therapy—previously noted for its safety in neuroblastoma—and found no objective responses in OS patients. However, a subset of patients experienced stable disease for 90 days with measurable CAR T cell expansion [42]. While these findings are encouraging, further optimization is needed before CAR T therapy becomes a reliable option for OS.

Another immunotherapeutic pathway under investigation is the CD47-SIRPα checkpoint, which regulates phagocytosis. Tumor cells can overexpress CD47 to mimic “self” signals, thereby avoiding destruction by macrophages. BMS-986351, a SIRPα-targeting antibody, has shown potential in enhancing macrophage-mediated phagocytosis, especially when combined with opsonizing antibodies. However, due to the widespread expression of SIRPα throughout the body, improving selectivity remains a key challenge [43]. A 2025 phase I/II trial evaluated the combination of margolimab (a CD47 inhibitor) and cetuximab (an EGFR blocker) in KRAS wild-type colorectal cancer. While not specific to OS, the combination showed tolerability and anti-tumor activity, suggesting CD47 blockade could have broader applicability [44].

Other immunotherapy strategies, such as cancer vaccines, are also under active investigation. These approaches—particularly those involving dendritic cell activation—have demonstrated variable results in both preclinical and clinical trials, highlighting the need for continued refinement [45]. A major challenge in immunotherapy remains treatment-related toxicity. Overactivation of the immune system can cause severe side effects, including gastrointestinal, endocrine, and dermatologic toxicity. More serious immune-related adverse events include neurotoxicity, cardiotoxicity, and pulmonary toxicity [46]. If significant toxicity develops, therapy is typically discontinued, and corticosteroids or immunosuppressive agents may be administered. These adverse effects are believed to stem from immune attacks on normal tissues that share molecular markers with tumor cells, resulting in autoimmune-like responses.

The TME plays a crucial role in immunotherapy resistance and effectiveness. In OS, the TME includes cancer cells, stromal components, blood vessels, soluble factors, and immune cells such as osteoblasts, osteoclasts, macrophages, and T cells. The TME is highly immunosuppressive due to molecules like TGF-β and IL-10, which inhibit immune responses and promote tumor growth [47, 48]. Additionally, TME-resident cells—including tumor-associated macrophages, NK cells, and regulatory T cells—can be reprogrammed to support immune evasion. Because many of these cells express checkpoint proteins such as PD-1 and CD47, they represent important targets for ongoing immunotherapy research [28].

Emerging studies in other cancers offer valuable insights. For example, research on the breast cancer TME has identified hub genes associated with CD8^+^ T cell exhaustion that correlate with disease progression and treatment outcomes [49]. Similar transcriptomic and functional analyses could be applied to tumor-associated macrophages or other immune cell types in OS, offering new avenues for targeted immunotherapy development.

### Radiation innovations

Traditionally, OS has been considered a radioresistant tumor, as many patients do not respond well to conventional radiotherapy. However, in select cases—such as craniofacial OS where surgery is not feasible—radiation therapy has shown some benefit in improving outcomes [50]. Traditional radiotherapy uses high-energy X-rays to destroy cancerous tissue. These X-rays are directed at the tumor, where the radiation damages DNA directly by breaking its strands, leading to cell death. Additionally, the radiation creates free radicals in the surrounding tissue, which further damage DNA and other cellular components [51]. A major drawback of conventional radiotherapy is its limited accuracy. This often results in collateral damage, affecting not only cancerous cells but also adjacent healthy tissues [52]. Such off-target effects can lead to additional complications and side effects for the patient. Recent innovations in radiotherapy aim to overcome these limitations. Several new approaches are under active investigation, with proton beam therapy and carbon ion radiotherapy (CIRT) showing particularly promising results [53].

Proton beam therapy seeks to address this limitation by utilizing protons instead of conventional X-rays, enabling highly precise targeting of cancerous tissue [54]. Proton beams are generated by a cyclotron or synchrotron and accelerated toward the tumor site. A key advantage of using protons is their energy-dependent depth of penetration, known as the Bragg peak. This phenomenon allows protons to deposit the majority of their energy precisely at the tumor site before dissipating, thereby minimizing damage to surrounding and downstream healthy tissues [55].

A notable limitation of proton beam therapy lies in the nonlinear travel path of protons in certain cases, which can reduce its precision [56]. At the end of the proton’s path, its relative biological effectiveness (RBE)—a reference measure compared to external radiation from cobalt-60 (^60^Co)—increases. This variability in RBE introduces uncertainty in dose distribution and diminishes the precision of treatment planning. As a result, proton beam therapy is generally avoided in cases where critical organs are located directly distal to the tumor site [55]. Despite this drawback, proton therapy remains significantly more effective and precise than traditional X-ray-based radiotherapy, offering improved outcomes by minimizing collateral damage to healthy tissues.

Similarly, CIRT—an advanced form of radiotherapy—offers even greater precision than traditional X-rays or proton therapy, owing to its use of carbon ions for targeted treatment [57]. Like proton beams, carbon ions exhibit a Bragg peak, allowing for focused energy deposition at the tumor site. Additionally, the molecular structure of carbon ions enables a higher rate of linear energy transfer (LET) compared to protons. This results in a greater RBE, allowing carbon ion therapy to inflict more substantial DNA damage on cancer cells than either proton beam therapy or conventional radiotherapy [58].

Despite promising results, the widespread use of both proton and CIRT remains limited to specialized facilities with substantial scientific and financial investment. These therapies require highly advanced and expensive equipment that is only available at select treatment centers. Due to the size and cost of the gantry systems used in CIRT, most centers rely on fixed-beam gantries. This setup limits the range of treatment angles and necessitates adjustments in patient positioning [58]. As research continues to validate the clinical benefits of these radiotherapy innovations, broader implementation is anticipated within the next four to five decades [59].

The differences in treatment cost between CIRT and PBT vary depending on the location of the tumor. Locations like the lung or prostate have a relatively similar cost per treatment, but locations like the head and neck will see larger differences [60]. Both treatments are still relatively expensive, averaging approximately $10,000–$40,000 for treatment, depending on the location, with areas like the head and neck reaching the higher ends [61]. Along with the higher cost, a major limiting factor in the implementation of either radiation method is the lack of centers containing the equipment necessary to do so. PBT centers have become much cheaper, costing around $25–$30 million [62]. In contrast, CIRT still remains very expensive, being estimated to cost around $300 million to develop a center capable of CIRT [63]. The high costs of centers capable of either of these treatments have stopped their implementation, with centers being concentrated mainly in the U.S, Europe, and Asia [62]. Despite the high costs, some cost-effectiveness analyses have found PBT especially to be cost-effective in the treatment of head and neck cancers—particularly in pediatric patients [64]. A separate study found that CIRT was a cost-effective option in the treatment of non-small-cell lung cancer, with high costs being incurred from unnecessary examinations and hospitalizations [65]. Use of PBT and CIRT was cost-effective in these cases because the treatment method lowered the chance for further complications following treatment, incurring a larger upfront cost but becoming cheaper over time [64, 65]. It has yet to be seen if PBT and CIRT will be cost-effective options for the treatment of patients with OS, but the limited accessibility due to high upfront costs and lack of centers capable of performing the treatments is likely to delay any such observations.

Another area of radiotherapy research that has been of high priority in recent years is research over radiosensitizers. Radiosensitizers increase the sensitivity of the cancer cells to radiation therapy without harming normal, healthy cells [66]. The mechanisms of action for these compounds are thought to be: (I) inhibiting radiation-induced repair of DNA damage, increasing the degree of DNA damage; (II) disturbing the cell cycle and organelle function to improve cytotoxicity; and (III) inhibiting the expression of radiation resistance genes, or promoting the expression of radiation-sensitive genes [67]. It has recently been found that organic compounds like ginseng polysaccharide (GPS) will increase the sensitivity of OS cells to ionizing radiotherapy [66]. It was discovered that GPS decreases the phosphorylation of p38 and AKT, which, when activated, play a major role in OS malignancies [60]. In addition, GPS decreases the anti-apoptosis protein Bcl2 and increases levels of the pro-apoptosis proteins Bax and cleaved-caspase3 [60]. In addition, several other naturally occurring compounds (paclitaxel, curcumin, genistein, papaverine, and resveratrol) are in clinical trials and have been shown to be effective radiosensitizers in independent studies [67]. Further research should be conducted to investigate the relationship between naturally found compounds such as these and the sensitivity of cancer cells to radiotherapy.

Some research regarding radiopharmaceuticals has undergone testing however, the literature is limited regarding effect on OS cancer cells.

### Tyrosine kinase inhibitors

Another area of science being heavily tested for treatment of OS is tyrosine kinase inhibitors (TKIs). For cases of OS where the cancerous cells are in an advanced stage, have metastasized, or are particularly resistant to other treatments, TKIs are particularly useful [68]. The principle in which this therapy operates is by targeting specific cellular pathways which are involved in tumor growth and metastasis [69]. They compete with ATP on the ATP binding site of tyrosine kinases, reducing tyrosine kinase phosphorylation and inhibiting its activity [70]. Most TKIs target multiple pathways, reducing the information available about which pathway is crucial for successful treatment [71], with the five most targeted receptors of TKIs being: KIT, vascular endothelial growth factor receptors (VEGFRs), RET, platelet-derived growth factor receptors (PDGFRs), and fibroblast growth factor receptor 1 (FGFR1) [72]. Another study has also shown that TKIs can increase the sensitivity of OS cells to chemotherapies by increasing the number of cells in the G2/M phase. This study found the effect in anlotinib however, other connections may be found with further research [73].

#### KIT

KIT is a type of RTK with a stem cell factor as its ligand. When bonding to its ligand, KIT triggers many signaling cascades that are critical to important cellular processes like differentiation, proliferation, and migration [72]. KIT has mostly been researched in non-oncological diseases; however, some studies have shown KIT expression to be evident in OSs [73]. Another study found that KIT-positive OSs were not as responsive to chemotherapy [71]. However, although many effective OS TKIs target KIT, there are ineffective TKIs that also target KIT, suggesting that targeting solely KIT is not an effective treatment for OS [71].

#### VEGFR

A common target of many TKI drugs is VEGF. Expression of VEGF and VEGFA in patients with OS has been linked to worse disease-free survival [74] and overall survival [75]. In IHC studies, VEGF expression was detected in 63–74% of OS samples. In another next generation sequencing (NGS) study, 41.2% of metastatic/recurrent samples were shown to be expressing VEGF [76]. Inhibition of this gene prevents angiogenesis, the formation of new blood vessels, and one of the six hallmarks of tumor formation [66]. Inhibiting angiogenesis will halt tumor growth and improve patient outcomes [77], and thus, VEGF is a critical target for TKIs in the treatment of OS.

#### FGFR

Another RTK often overexpressed in patients with OS is FGFR [78]. FGFRs regulate nervous system control, organogenesis, and tissue repair, along with others [79]. FGFR1 is expressed in 74% of OS samples, making it another prime target for TKIs [78].

#### PDGFR & mesenchymal-epithelial transition

TKIs also include mesenchymal-epithelial transition (MET) factor and PDGFR, which are involved in pathways important for tumor growth and proliferation. Overproduction of MET in patients diagnosed with OS tends to result in poorer patient outcomes as well as an increased chance of metastasis [80]. Following a similar mechanism, PDGFR plays a role in various biological processes, including cell growth, survival, migration, and differentiation [81]. It is part of a family of receptors that bind to PDGFs, which are secreted proteins involved in signaling within the TME and stromal cells. Many TKIs that are undergoing clinical testing aim to target both MET and PDGFR [82]. Drugs like cabozantinib, regorafenib, and pazopanib target both MET and PDGFR pathways, making them valuable for OS cases with dual pathway dysregulation [83].

#### RET

RET is another, less studied RTK involved in OS. Thought to be a promoter of metastatic behavior, as well as being associated with increased chemotherapeutic resistance, RET is a target of many TKIs [84]. RET has also been associated with an increase in stem cell-like properties of the OS [72]. RET thus is a critical target in the future of TKI treatment and needs further study.

#### Insulin-like growth factor receptor

Although not one of the most targeted pathways, IGF-R (insulin-like growth factor receptor) has been implicated in promoting cell growth and inhibiting apoptosis, allowing for tumors to grow, mainly through the PI3K and MAPK pathways [85]. In addition, expression of IGF-1R was found to be closely associated with metastasis in OS patients [86]. A review from Moukengue et al. [87] cited that IGF expression was linked with increased OS aggression, increased distant metastasis, and decreased overall rates of survival.

#### AXL

AXL has been found to be highly expressed in OS and is positively correlated with negative prognosis [88]. A study done in murine models discovered that AXL inhibition significantly reduced the number of MG63.2 pulmonary metastases [89]. In clinical trials done using multi-target TKIs, when AXL was inhibited, some patients showed positive remission [66]. Although this is a promising result, no single-target study has been done on the effects of inhibiting AXL and patient outcomes. In most OS tissues, AXL is highly expressed. Its knockdown has been correlated to inhibition of proliferation and inducement of apoptosis of OS cells [90]. A preclinical trial also found that AXL was overexpressed in a highly metastatic OS cell line and that its inhibition reduced cell proliferation, invasion, and metastasis [88].

#### Overview

The success of these therapies in smaller trials has shown effectiveness for OS treatment however, broader use would depend on the results of phase 3 trials that confirm their efficacy compared to current standard therapies. By simultaneously inhibiting angiogenesis, tumor cell proliferation, and metastasis-related pathways, these agents can potentially improve outcomes in aggressive and metastatic OS [91]. Although these drugs can also inhibit multiple targets, they may have an unknown impact on the patient [71]. Some studies have reported that TKIs have affected multiple organs in the body, or may have cardiovascular side effects such as hypertension, atrial fibrillation, reduced cardiac function, and heart failure, to sudden death [84]. A recent review by Shyam Sunder et al. [91] provides a more extensive overview of these adverse cardiovascular effects and their proposed mechanisms.

## Analysis

This review has highlighted the complex biology of OS, including the roles of genetic mutations, the TME, and angiogenesis in disease progression. It has also examined the current treatment landscape and explored emerging therapeutic strategies. Despite significant advancements, OS remains a challenging malignancy to treat, with high rates of metastasis and recurrence. Many questions remain unanswered, particularly regarding the molecular mechanisms underlying therapy resistance. Collaboration among researchers, clinicians, and biotechnology companies will be essential to accelerating the development of effective treatments. With continued research and technological progress, there is hope for more targeted therapies and improved survival outcomes for patients with OS.

## Abbreviations

CAR: chimeric antigen receptor
CIRT: carbon ion radiotherapy
FGFR: fibroblast growth factor receptor
GPS: ginseng polysaccharide
IGF-R: insulin-like growth factor receptor
MET: mesenchymal-epithelial transition
OS: osteosarcoma
PDGFRs: platelet-derived growth factor receptors
pegRNA: prime editing guide RNA
RBE: relative biological effectiveness
TKIs: tyrosine kinase inhibitors
TME: tumor microenvironment
VEGFRs: vascular endothelial growth factor receptors

## Declarations

### Copyright

© The Author(s) 2025.

## References

[1] Sadykova LR, Ntekim AI, Muyangwa-Semenova M, Rutland CS, Jeyapalan JN, Blatt N (2020). Epidemiology and Risk Factors of Osteosarcoma. Cancer Invest.

[2] Chen G, Li M, Xiao X, Ji C, Huang M, Wang Z (2023). A classification system of joint-salvage tumor resection in osteosarcoma of the knee: A retrospective cohort study. Knee.

[3] Cortini M, Avnet S, Baldini N (2017). Mesenchymal stroma: role in osteosarcoma progression. Cancer Lett.

[4] Abarrategi A, Tornin J, Martinez-Cruzado L, Hamilton A, Martinez-Campos E, Rodrigo JP (2016). Osteosarcoma: Cells-of-Origin, Cancer Stem Cells, and Targeted Therapies. Stem Cells Int.

[5] Deepak KV, Bharanidharan R (2025). A survey on deep learning and machine learning techniques over histopathology image based Osteosarcoma Detection. Multimed Tools Appl.

[6] Jeffree GM, Price CH, Sissons HA (1975). The metastatic patterns of osteosarcoma. Br J Cancer.

[7] Mohseny AB, Szuhai K, Romeo S, Buddingh EP, Briaire-de Bruijn I, de Jong D (2009). Osteosarcoma originates from mesenchymal stem cells in consequence of aneuploidization and genomic loss of *Cdkn2*. J Pathol.

[8] Marko TA, Diessner BJ, Spector LG (2016). Prevalence of Metastasis at Diagnosis of Osteosarcoma: An International Comparison. Pediatr Blood Cancer.

[9] Harper K, Sathiadoss P, Saifuddin A, Sheikh A (2021). A review of imaging of surface sarcomas of bone. Skeletal Radiol.

[10] Lindsey BA, Markel JE, Kleinerman ES (2017). Osteosarcoma Overview. Rheumatol Ther.

[11] Buchanan A, Abdelsayed R, Kalathingal S, Kurago Z (2021). Radiographic features of intercrestal and classic osteosarcoma: a report of 3 cases. Or Surg Or Med Or Pa Or Radiol.

[12] Cleton-Jansen AM, Buerger H, Hogendoorn PCW (2005). Central high-grade osteosarcoma of bone: diagnostic and genetic considerations. Curr Diagn Pathol.

[13] Kumar VS, Barwar N, Khan SA (2014). Surface osteosarcomas: Diagnosis, treatment and outcome. Indian J Orthop.

[14] Hang JF, Chen PC (2014). Parosteal Osteosarcoma. Arch Pathol Lab Med.

[15] Cesari M, Alberghini M, Vanel D, Palmerini E, Staals EL, Longhi A (2011). Periosteal osteosarcoma: A single-institution experience. Cancer.

[16] Deng Z, Huang Z, Ding Y, Su Y, Chan CM, Niu X (2020). High-Grade Surface Osteosarcoma: Clinical Features and Oncologic Outcome. J Bone Oncol.

[17] Beird HC, Bielack SS, Flanagan AM, Gill J, Heymann D, Janeway KA (2022). Osteosarcoma. Nat Rev Dis Primers.

[18] Crombé A, Simonetti M, Longhi A, Hauger O, Fadli D, Spinnato P (2024). Imaging of Osteosarcoma: Presenting Findings, Metastatic Patterns, and Features Related to Prognosis. J Clin Med.

[19] Barger A, Baker K, Driskell E, Sander W, Roady P, Berry M (2022). The use of alkaline phosphatase and runx2 to distinguish osteosarcoma from other common malignant primary bone tumors in dogs. Vet Pathol.

[20] Lin T, Jin Q, Mo X, Zhao Z, Xie X, Zou C (2021). Experience with periprosthetic infection after limb salvage surgery for patients with osteosarcoma. J Orthop Surg Res.

[21] Marchandet L, Lallier M, Charrier C, Baud’huin M, Ory B, Lamoureux F (2021). Mechanisms of Resistance to Conventional Therapies for Osteosarcoma. Cancers (Basel).

[22] Benjamin RS (2020). Adjuvant and Neoadjuvant Chemotherapy for Osteosarcoma: A Historical Perspective. Adv Exp Med Biol.

[23] Papakonstantinou E, Stamatopoulos A, I Athanasiadis D, Kenanidis E, Potoupnis M, Haidich A (2020). Limb-salvage surgery offers better five-year survival rate than amputation in patients with limb osteosarcoma treated with neoadjuvant chemotherapy. A systematic review and meta-analysis. J Bone Oncol.

[24] Jafari F, Javdansirat S, Sanaie S, Naseri A, Shamekh A, Rostamzadeh D (2020). Osteosarcoma: A comprehensive review of management and treatment strategies. Ann Diagn Pathol.

[25] Yan JP, Xiang RM (2021). Effect assessment of methotrexate in combination with other chemotherapeutic agents for osteosarcoma in children: A protocol for systematic review and meta-analysis. Medicine (Baltimore).

[26] Rothzerg E, Pfaff AL, Koks S (2022). Innovative approaches for treatment of osteosarcoma. Exp Biol Med (Maywood).

[27] Celik B, Cicek K, Leal AF, Tomatsu S (2022). Regulation of Molecular Targets in Osteosarcoma Treatment. Int J Mol Sci.

[28] Yu S, Yao X (2024). Advances on immunotherapy for osteosarcoma. Mol Cancer.

[29] Fu H, Wu Y, Chen J, Hu X, Wang X, Xu G (2023). Exosomes and osteosarcoma drug resistance. Front Oncol.

[30] Théry C, Amigorena S, Raposo G, Clayton A (2006). Isolation and Characterization of Exosomes from Cell Culture Supernatants and Biological Fluids. Curr Protoc Cell Biol.

[31] Zhang B, Yang Y, Tao R, Yao C, Zhou Z, Zhang Y (2022). Exosomes loaded with miR-665 inhibit the progression of osteosarcoma *in vivo* and *in vitro*. Am J Transl Res.

[32] Saber Sichani A, Ranjbar M, Baneshi M, Torabi Zadeh F, Fallahi J (2023). A Review on Advanced CRISPR-Based Genome-Editing Tools: Base Editing and Prime Editing. Mol Biotechnol.

[33] Liu W, Wang S, Lin B, Zhang W, Ji G (2021). Applications of CRISPR/Cas9 in the research of malignant musculoskeletal tumors. BMC Musculoskelet Disord.

[34] Chehelgerdi M, Chehelgerdi M, Khorramian-Ghahfarokhi M, Shafieizadeh M, Mahmoudi E, Eskandari F (2024). Comprehensive review of CRISPR-based gene editing: mechanisms, challenges, and applications in cancer therapy. Mol Cancer.

[35] Wang R, Wang D, Bai X, Guo J, Xia S, Cheng Y (2023). Kinome-wide CRISPR-Cas9 knockout screens revealed *PLK1* as a therapeutic target for osteosarcoma. Cell Death Discov.

[36] Wang J, Guo W, Wang X, Tang X, Sun X, Ren T (2023). Circulating Exosomal PD-L1 at Initial Diagnosis Predicts Outcome and Survival of Patients with Osteosarcoma. Clin Cancer Res.

[37] Yu X, Zhai X, Wu J, Feng Q, Hu C, Zhu L (2024). Evolving perspectives regarding the role of the PD-1/PD-L1 pathway in gastric cancer immunotherapy. Biochim Biophys Acta Mol Basis Dis.

[38] Boye K, Longhi A, Guren T, Lorenz S, Næss S, Pierini M (2021). Pembrolizumab in advanced osteosarcoma: results of a single-arm, open-label, phase 2 trial. Cancer Immunol Immunother.

[39] Park JA, Cheung NV (2023). Promise and Challenges of T Cell Immunotherapy for Osteosarcoma. Int J Mol Sci.

[40] Curigliano G, Gelderblom H, Mach N, Doi T, Tai D, Forde PM (2021). Phase I/Ib Clinical Trial of Sabatolimab, an Anti-TIM-3 Antibody, Alone and in Combination with Spartalizumab, an Anti-PD-1 Antibody, in Advanced Solid Tumors. Clin Cancer Res.

[41] Khan SH, Choi Y, Veena M, Lee JK, Shin DS (2025). Advances in CAR T cell therapy: antigen selection, modifications, and current trials for solid tumors. Front Immunol.

[42] Kaczanowska S, Murty T, Alimadadi A, Contreras CF, Duault C, Subrahmanyam PB (2024). Immune determinants of CAR-T cell expansion in solid tumor patients receiving GD2 CAR-T cell therapy. Cancer Cell.

[43] Chan H, Trout CV, Mikolon D, Adams P, Guzman R, Mavrommatis K (2024). Discovery and Preclinical Activity of BMS-986351, an Antibody to SIRPα That Enhances Macrophage-mediated Tumor Phagocytosis When Combined with Opsonizing Antibodies. Cancer Res Commun.

[44] Eng C, Lakhani NJ, Philip PA, Schneider C, Johnson B, Kardosh A (2025). A Phase 1b/2 Study of the Anti-CD47 Antibody Magrolimab with Cetuximab in Patients with Colorectal Cancer and Other Solid Tumors. Target Oncol.

[45] Musser ML, Berger EP, Tripp CD, Clifford CA, Bergman PJ, Johannes CM (2021). Safety evaluation of the canine osteosarcoma vaccine, live *Listeria* vector. Vet Comp Oncol.

[46] Choi J, Lee SY (2020). Clinical Characteristics and Treatment of Immune-Related Adverse Events of Immune Checkpoint Inhibitors. Immune Netw.

[47] Tian B, Du X, Zheng S, Zhang Y (2022). The Role of Tumor Microenvironment in Regulating the Plasticity of Osteosarcoma Cells. Int J Mol Sci.

[48] Han Z, Chen G, Wang D (2025). Emerging immunotherapies in osteosarcoma: from checkpoint blockade to cellular therapies. Front Immunol.

[49] Liu H, Dong A, Rasteh AM, Wang P, Weng J (2024). Identification of the novel exhausted T cell CD8^+^ markers in breast cancer. Sci Rep.

[50] Wu Y, Song Y, Wang R, Wang T (2023). Molecular mechanisms of tumor resistance to radiotherapy. Mol Cancer.

[51] Dong M, Liu R, Zhang Q, Luo H, Wang D, Wang Y (2022). Efficacy and safety of carbon ion radiotherapy for bone sarcomas: a systematic review and meta-analysis. Radiat Oncol.

[52] Seidensaal K, Mattke M, Haufe S, Rathke H, Haberkorn U, Bougatf N (2021). The role of combined ion-beam radiotherapy (CIBRT) with protons and carbon ions in a multimodal treatment strategy of inoperable osteosarcoma. Radiother Oncol.

[53] Astl J, Belsan T, Michnova L, Kubeš J, Filipovsky T, Blecha J (2022). Highly Aggressive Osteosarcoma of the Ethmoids and Maxillary Sinus-A Case of Successful Surgery and Proton Beam Radiotherapy in a 65-Year-Old Man. Medicina (Kaunas).

[54] Tian X, Liu K, Hou Y, Cheng J, Zhang J (2018). The evolution of proton beam therapy: Current and future status. Mol Clin Oncol.

[55] Mehta R, Ponnuvelu S, Kuotsu R, Nagarkar NM (2023). Osteosarcoma of the sphenoid sinus extending to ethmoid sinus—report of a rare case with review of literature giving special emphasis on treatment and outcome. Egypt J Otolaryngol.

[56] Hoppe BS, Petersen IA, Wilke BK, DeWees TA, Imai R, Hug EB (2023). Pragmatic, Prospective Comparative Effectiveness Trial of Carbon Ion Therapy, Surgery, and Proton Therapy for the Management of Pelvic Sarcomas (Soft Tissue/Bone) Involving the Bone: The PROSPER Study Rationale and Design. Cancers (Basel).

[57] Malouff TD, Mahajan A, Krishnan S, Beltran C, Seneviratne DS, Trifiletti DM (2020). Carbon Ion Therapy: A Modern Review of an Emerging Technology. Front Oncol.

[58] Kowalchuk RO, Corbin KS, Jimenez RB (2022). Particle Therapy for Breast Cancer. Cancers (Basel).

[59] Zhao X, Wu Q, Gong X, Liu J, Ma Y (2021). Osteosarcoma: a review of current and future therapeutic approaches. Biomed Eng Online.

[60] Zhang XY, Sun K, Zhu Q, Song T, Liu Y (2017). Ginseng polysaccharide serves as a potential radiosensitizer through inducing apoptosis and autophagy in the treatment of osteosarcoma. Kaohsiung J Med Sci.

[61] Peeters A, Grutters JPC, Pijls-Johannesma M, Reimoser S, De Ruysscher D, Severens JL (2010). How costly is particle therapy? Cost analysis of external beam radiotherapy with carbon-ions, protons and photons. Radiother Oncol.

[62] Gaito S, Aznar MC, Burnet NG, Crellin A, France A, Indelicato D (2023). Assessing Equity of Access to Proton Beam Therapy: A Literature Review. Clin Oncol (R Coll Radiol).

[63] Beltran C, Amos RA, Rong Y (2020). We are ready for clinical implementation of Carbon Ion Radiotherapy in the United States. J Appl Clin Med Phys.

[64] Bharathi RP, Ms A, Kamath A (2023). A Systematic Review of the Economic Burden of Proton Therapy in Head and Neck Cancer. Asian Pac J Cancer Prev.

[65] Okazaki S, Shibuya K, Takura T, Miyasaka Y, Kawamura H, Ohno T (2022). Cost-effectiveness of carbon-ion radiotherapy versus stereotactic body radiotherapy for non-small-cell lung cancer. Cancer Sci.

[66] Komorowska D, Radzik T, Kalenik S, Rodacka A (2022). Natural Radiosensitizers in Radiotherapy: Cancer Treatment by Combining Ionizing Radiation with Resveratrol. Int J Mol Sci.

[67] Giordano F, Lenna S, Baudo G, Rampado R, Massaro M, De Rosa E (2022). Tyrosine kinase inhibitor-loaded biomimetic nanoparticles as a treatment for osteosarcoma. Cancer Nano.

[68] Sheng G, Gao Y, Yang Y, Wu H (2021). Osteosarcoma and Metastasis. Front Oncol.

[69] Jiao Q, Bi L, Ren Y, Song S, Wang Q, Wang YS (2018). Advances in studies of tyrosine kinase inhibitors and their acquired resistance. Mol Cancer.

[70] Kyriazoglou A, Gkaralea LE, Kotsantis I, Anastasiou M, Pantazopoulos A, Prevezanou M (2022). Tyrosine kinase inhibitors in sarcoma treatment. Oncol Lett.

[71] Tian Z, Niu X, Yao W (2020). Receptor Tyrosine Kinases in Osteosarcoma Treatment: Which Is the Key Target?. Front Oncol.

[72] Wang G, Sun M, Jiang Y, Zhang T, Sun W, Wang H (2019). Anlotinib, a novel small molecular tyrosine kinase inhibitor, suppresses growth and metastasis *via* dual blockade of VEGFR2 and MET in osteosarcoma. Int J Cancer.

[73] Yang J, Yang D, Sun Y, Sun B, Wang G, Trent JC (2011). Genetic amplification of the vascular endothelial growth factor (VEGF) pathway genes, including *VEGFA*, in human osteosarcoma. Cancer.

[74] Yu XW, Wu TY, Yi X, Ren WP, Zhou ZB, Sun YQ (2014). Prognostic significance of VEGF expression in osteosarcoma: a meta-analysis. Tumour Biol.

[75] Suehara Y, Alex D, Bowman A, Middha S, Zehir A, Chakravarty D (2019). Clinical Genomic Sequencing of Pediatric and Adult Osteosarcoma Reveals Distinct Molecular Subsets with Potentially Targetable Alterations. Clin Cancer Res.

[76] Assi T, Watson S, Samra B, Rassy E, Le Cesne A, Italiano A (2021). Targeting the VEGF Pathway in Osteosarcoma. Cells.

[77] Assi A, Farhat M, Hachem MCR, Zalaquett Z, Aoun M, Daher M (2023). Tyrosine kinase inhibitors in osteosarcoma: Adapting treatment strategiesa. J Bone Oncol.

[78] Imamura T (2014). Physiological Functions and Underlying Mechanisms of Fibroblast Growth Factor (FGF) Family Members: Recent Findings and Implications for Their Pharmacological Application. Biol Pharm Bull.

[79] Yu X, Yustein JT, Xu J (2021). Research models and mesenchymal/epithelial plasticity of osteosarcoma. Cell Biosci.

[80] Chen X, Liu L, Liu P, Chen Y, Lin D, Yan H (2022). Discovery of Potent and Orally Bioavailable Platelet-Derived Growth Factor Receptor (PDGFR) Inhibitors for the Treatment of Osteosarcoma. J Med Chem.

[81] Zvi Y, Ugur E, Batko B, Gill J, Roth M, Gorlick R (2021). Prognostic and Therapeutic Utility of Variably Expressed Cell Surface Receptors in Osteosarcoma. Sarcoma.

[82] Wang JH, Zeng Z, Sun J, Chen Y, Gao X (2021). A novel small-molecule antagonist enhances the sensitivity of osteosarcoma to cabozantinib in vitro and in vivo by targeting DNMT-1 correlated with disease severity in human patients. Pharmacol Res.

[83] Luo J, Xia Y, Yin Y, Luo J, Liu M, Zhang H (2019). ATF4 destabilizes RET through nonclassical GRP78 inhibition to enhance chemosensitivity to bortezomib in human osteosarcoma. Theranostics.

[84] Ji Z, Shen J, Lan Y, Yi Q, Liu H (2023). Targeting signaling pathways in osteosarcoma: Mechanisms and clinical studies. MedComm (2020).

[85] Wang YH, Han XD, Qiu Y, Xiong J, Yu Y, Wang B (2012). Increased expression of insulin-like growth factor-1 receptor is correlated with tumor metastasis and prognosis in patients with osteosarcoma. J Surg Oncol.

[86] Li Q, Wang X, Jiang N, Xie X, Liu N, Liu J (2020). Exosome-transmitted linc00852 associated with receptor tyrosine kinase AXL dysregulates the proliferation and invasion of osteosarcoma. Cancer Med.

[87] Moukengue B, Lallier M, Marchandet L, Baud’huin M, Verrecchia F, Ory B (2022). Origin and Therapies of Osteosarcoma. Cancers (Basel).

[88] Lamhamedi-Cherradi SE, Mohiuddin S, Mishra DK, Krishnan S, Velasco AR, Vetter AM (2021). Transcriptional activators YAP/TAZ and AXL orchestrate dedifferentiation, cell fate, and metastasis in human osteosarcoma. Cancer Gene Ther.

[89] Just MA, Van Mater D, Wagner LM (2021). Receptor tyrosine kinase inhibitors for the treatment of osteosarcoma and Ewing sarcoma. Pediatr Blood Cancer.

[90] Nirala BK, Yamamichi T, Yustein JT (2023). Deciphering the Signaling Mechanisms of Osteosarcoma Tumorigenesis. Int J Mol Sci.

[91] Shyam Sunder S, Sharma UC, Pokharel S (2023). Adverse effects of tyrosine kinase inhibitors in cancer therapy: pathophysiology, mechanisms and clinical management. Signal Transduct Target Ther.

